# Selective expression of muscarinic acetylcholine receptor subtype M3 by mouse type III taste bud cells

**DOI:** 10.1007/s00424-016-1879-5

**Published:** 2016-09-14

**Authors:** Yusuke Mori, Kohgaku Eguchi, Kiyonori Yoshii, Yoshitaka Ohtubo

**Affiliations:** Graduate School of Life Science and Systems Engineering, Kyushu Institute of Technology, Kitakyushu, 808-0196 Japan

**Keywords:** Ca^2+^ imaging, Fungiform taste bud, M3 muscarinic acetylcholine receptor, Peeled lingual epithelia, Immunohistostaining

## Abstract

Each taste bud cell (TBC) type responds to a different taste. Previously, we showed that an unidentified cell type(s) functionally expresses a muscarinic acetylcholine (ACh) receptor subtype, M3, and we suggested the ACh-dependent modification of its taste responsiveness. In this study, we found that M3 is expressed by type III TBCs, which is the only cell type that possesses synaptic contacts with taste nerve fibers in taste buds. The application of ACh to the basolateral membrane of mouse fungiform TBCs in situ increased the intracellular Ca^2+^ concentration in 2.4 ± 1.4 cells per taste bud (mean ± SD, *n* = 14). After Ca^2+^ imaging, we supravitally labeled type II cells (phospholipase C β2 [PLCβ2]-immunoreactive cells) with Lucifer yellow CH (LY), a fluorescent dye and investigated the positional relationship between ACh-responding cells and LY-labeled cells. After fixation, the TBCs were immunohistostained to investigate the positional relationships between immunohistochemically classified cells and LY-labeled cells. The overlay of the two positional relationships obtained by superimposing the LY-labeled cells showed that all of the ACh-responding cells were type III cells (synaptosomal-associated protein 25 [SNAP-25]-immunoreactive cells). The ACh responses required no added Ca^2+^ in the bathing solution. The addition of 1 μM U73122, a phospholipase C inhibitor, decreased the magnitude of the ACh response, whereas that of 1 μM U73343, a negative control, had no effect. These results suggest that type III cells respond to ACh and release Ca^2+^ from intracellular stores. We also discuss the underlying mechanism of the Ca^2+^ response and the role of M3 in type III cells.

## Introduction

The responsiveness of taste bud cells (TBCs) depends on their cell types. Type II cells detect sweet, bitter, and umami substances [2, 3, 21, 23, 25, 26], and type III cells are considered to sense acidic substances [14, 17, 36] as well as mediate the taste responses of type II cells to the brain [4, 10, 13].

TBCs express neurotransmitter receptors for acetylcholine (ACh), ATP, serotonin, adrenaline, and cholecystokinin [5, 8, 10–12, 16, 18, 19, 27]. In our previous study, we showed that unidentified TBCs expressed a muscarinic ACh receptor subtype, M3, and these TBCs exhibited increased intracellular Ca^2+^ concentrations in response to ACh [8]. If the expression of M3 depends on the cell type, then the ACh released in taste bud selectively modifies the response according to the cell type. Therefore, we focused on the identification of cell type which functionally expresses M3.

In the present study, we identified the cell type that functionally expresses M3 in situ. As the antibodies against mouse M3 for immunohistochemical uses were unavailable to us, we employed Lucifer yellow CH (LY)-labeled type II cells under supravital staining conditions [32]. Thus, we labeled type II cells with LY after identifying ACh-responding cells by Ca^2+^ imaging and investigated the positional relationships between the LY-labeled cells and the ACh-responding cells. Next, we fixed TBCs and immunohistochemically classified the cells according to three types—type II cells, type III cells, and non-immunoreactive cells—before investigating the positional relationships between LY-labeled cells and each cell type in the taste bud. The overlay of the two positional relationships obtained by superimposing the LY-labeled cells identified the cell type of the ACh-responding cells. Thus, we show that M3 is selectively expressed by type III cells. We discuss the underlying mechanism that allows M3 to elicit Ca^2+^ responses and the role of M3 in taste responses.

### Preparation of peeled lingual epithelia

We killed ca. 6-week-old ddY strain mice of either sex by exposure to CO_2_ followed by decapitation. We removed their tongues and hypodermically injected an elastase solution into the tongue, before peeling the lingual epithelia containing fungiform taste buds. The peeled epithelium was mounted on a recording platform with the basolateral membrane side facing upward and then placed under a 60× water immersion objective (Fluor-60×, Olympus, Tokyo, Japan), as described previously [8, 10, 20]. The basolateral membrane side of the peeled epithelium was irrigated with either a control bathing solution or a test solution by exchanging the composition of the water column between the water immersion objective and the basolateral membrane side. The receptor membrane side facing inside the platform was allowed to acclimate to the control bathing solution.

### Ca^2+^ imaging

We also examined the [Ca^2+^]_in_ of TBCs in response to ACh, as described previously [8, 10], except we used another charge-coupled device (CCD) camera (C9100-13, Hamamatsu Photonics, Hamamatsu, Japan) to lower the noise and increase the resolution from 12-bit to 16-bit to clarify the outline of the ACh-sensitive TBCs. In brief, we soaked the basolateral membrane side of the peeled epithelium mounted on the recording platform in Fura-2 AM solution, which was then placed under a fluorescent microscope equipped with the water immersion objective lens. The Fura-2-stained TBCs were excited at 340 nm (F340) and 380 nm (F380) with a spectroscope-type high-speed wavelength changer (C7773, Hamamatsu Photonics). Images of Fura-2 fluorescence were acquired every 2.5 s using the CCD camera through the water immersion objective, stored in a computer, and analyzed with AQUACOSMOS software (version 2.6, Hamamatsu Photonics). We sequentially plotted the averaged [Ca^2+^]_in_ over the respective cell areas as the ratio of F340/F380, and we took the peak magnitude of the ratio as the response magnitude. The response magnitude was normalized against that elicited by 1 μM ACh dissolved in the control bathing solution, and we expressed the normalized response magnitude as the mean ± SD unless stated otherwise. We also plotted the number of ACh-responding cells as the mean ± SD.

### LY labeling without fixation

After the Ca^2+^ imaging study, we supravitally labeled type II cells in the taste buds with LY, as described previously [32]. In brief, we acclimated the basolateral membrane of TBCs in the peeled lingual epithelium to the control bathing solution under the microscope, before applying 200 mM K^+^ bathing solution containing 0.4 mg/ml LY to the basolateral membrane for 2 min in the dark, washing with the control bathing solution, and capturing a fluorescence image of the LY-labeled cells with the CCD camera. Care was taken to prevent the taste bud from moving, which was essential for investigating the positional relationships between ACh-responding cells and LY-labeled cells.

### Immunohistochemistry

After Ca^2+^ imaging and LY labeling, we fixed and immunohistostained the TBCs to investigate the positional relationships between the LY-labeled cells and each immunohistochemical cell type. It should be noted that the fluorescence of LY was still very clear after immunohistostaining.

The method employed for immunostaining and obtaining fluorescent images was similar to that used in our previous studies [8, 10, 29, 30]. In brief, the peeled lingual epithelium was fixed with paraformaldehyde solution at 4 °C, incubated in blocking solution containing 3 % normal goat serum, 0.3 % Triton X, and 1 % bovine serum albumin in phosphate-buffered saline (PBS), incubated with primary antibodies dissolved in the blocking solution at 4 °C for 24–48 h, rinsed with PBS, and incubated with Alexa Fluor-conjugated secondary antibodies dissolved in the blocking solution at 4 °C for 24–48 h. The epithelium was mounted on glass slides and then sliced optically and horizontally to obtain fluorescence images of the immunostained and LY-labeled TBCs with a laser scanning confocal microscope system (Leica Microsystems, Bensheim, Germany). It should be noted that the fluorescence due to immunoreactivity and LY was captured in the same focal plane.

The primary antibodies were anti-SNAP-25 mouse monoclonal antibody (1:1000; Sigma, St. Louis, MO, USA) and anti-PLCβ2 rabbit antibody (1:100; Santa Cruz Biotechnology). The secondary antibodies were Alexa Fluor 488-conjugated goat anti-rabbit IgG (1:400; Molecular Probes, Eugene, OR, USA) and Alexa Fluor 555-conjugated goat anti-mouse IgG (1:400; Molecular Probes).

### Multiplex nested reverse transcription polymerase chain reaction

We searched for messenger RNAs (mRNAs) encoding enzymes involved in IP_3_ cascades using mouse fungiform taste buds isolated from peeled lingual epithelia, as described previously [8, 10]. In brief, we soaked the peeled lingual epithelium in a nominally Ca^2+^- and Mg^2+^-free solution for ca 1 min, puffed the nominally Ca^2+^- and Mg^2+^-free solution through a pipette onto the cleft between a taste bud and the surrounding epithelium cells in the control bathing solution under the microscope, and then collected the liberated taste bud with another suction pipette. The taste buds were collected and used in the PCR experiments describe as follows:

We employed multiplex nested reverse transcription polymerase chain reaction (RT-PCR), which comprises two steps (first RT-PCR and second PCR), thereby reducing the number of mice sacrificed. The first RT-PCR was performed using a OneStep RT-PCR Kit (Qiagen, Valencia, CA). Three taste buds were added to a RT-PCR mix containing RNase inhibitor (Takara Bio Inc., Siga, Japan) and 0.6 μM outer primers (Table 1). The RT-PCR conditions were as follows: reverse transcription, 50 °C for 30 min; initial PCR activation step, 95 °C for 15 min; denaturation, 40 cycles at 94 °C for 30 s; annealing, 58 °C for 1 min; extension, 72 °C for 1.5 min; and final extension, 72 °C for 10 min. The amplified products (2 μl) were added to the second PCR mix containing DNA polymerase KOD plus (Toyobo Co. Ltd., Osaka, Japan) and 0.6 μM inner primers (Table 1). The conditions for the second round of PCR were as follows: pre-denaturation, 94 °C for 2 min; denaturation, 40 cycles at 94 °C for 30 s; annealing, 58 °C for 30 s; extension, 68 °C for 1 min; and final extension, 68 °C for 10 min. The PCR products were analyzed by 2 % agarose gel electrophoresis, stained with ethidium bromide (0.1 μg/ml), and visualized by ultraviolet illumination.

**Table 1:** Sequences of primer sets used for multiplex nested RT-PCR

Protein		Forward primer 5′–3′	Reverse primer 5′–3′	Product size (bp)
PLCβ2	Outer	AGCCTAAGCTTCCTCTCCTG	AGGAGTTGAGTCGAGGGTCT	*865*
	Inner	CTCGCTTTGGGAAGTTTGC	GCATTGACTGTCATCGGGT	*226*
PLCβ3	Outer	TCAAGGTCTGGTCAGAGGAG	GATGGCCTCTAGCACATCAC	*806*
	Inner	GGAGCAGCTCATGGACTTTA	CATACATCCAGCTCCACACA	*369*
IP_3_R3	Outer	GTCTGTAACAAGCGGGAGAA	GACTTGCGGTTCTGGAGATA	*749*
	Inner	CCAACCAGTGGGACTACAAG	CGAACTTGCTCTTCTTCAGC	*291*
IP_3_R1	Outer	CCTGTCTTTGTGCAACTCCT	CTCCTTGTCGAGGTGACACT	*888*
	Inner	CATAGCCATTCCTGTTGACC	CTCCAGCAGTTGCTTGGTAT	*352*
SNAP-25	Outer	AGGAAGGGATGGACCAAATC	GGGGGTGACTGACTCTGTGT	*600*
	Inner	GGCAATAATCAGGATGGAGTAG	AAATTTAACCACTTCCCAGCA	*310*
NTPD2	Outer	CCTCAAGTATGGCATCGTTC	TATTGAAGAGCCCAGAGACG	*848*
	Inner	GTGACTGCCAACTACCTGCT	GACCCATAGTGCATGGAGAC	*352*

Each outer primer set was mixed in a single tube containing the collected taste buds and reagents for one-step RT-PCR. The amplicon and inner primer sets were used for second PCR in individual reaction tubes

The optimum annealing temperatures were determined in preliminary PCR experiments using brain tissue and peeled lingual epithelium containing TBCs. We tested a range of annealing temperatures from 50 to 62 °C in 4 °C steps, and we selected the annealing temperature that yielded a clear single band with the correct size on agarose gels for each primer set. As cell type markers, we used nucleoside triphosphate diphosphohydrolase 2 (NTPD2) for type I cells; phospholipase C β2 (PLCβ2) and type III inositol 1,4,5-triphosphate receptor (IP_3_R3) for type II cells; and synaptosomal-associated protein 25 (SNAP-25) for type III cells. The primers were obtained from Genenet (Fukuoka, Japan).

### Solutions

Unless stated otherwise, all of the reagents were obtained from Wako (Osaka, Japan). All of the solutions were prepared with deionized water and the components were expressed in millimolar concentrations, unless stated otherwise.

The control bathing solution comprised 150 NaCl, 5 KCl, 2 CaCl_2_, 0.5 MgCl_2_, 10 glucose, and 5 HEPES (Sigma, St. Louis, MO), which was buffered to pH 7.4 with NaOH. The 200 mM K^+^ bathing solution was prepared by increasing the KCl concentration to 200 mM and eliminating NaCl from the control bathing solution. The 50 mM K^+^ bathing solution was prepared by replacing isomolar Na^+^ in the control bathing solution. The nominally Ca^2+^-free bathing solution was prepared by replacing CaCl_2_ with MgCl_2_ in the control bathing solution. Earle’s solution comprised 116 NaCl, 5.4 KCl, 1.8 CaCl_2_, 0.8 MgSO_4_, 26.2 NaHCO_3_, and 1 NaH_2_PO_4_. PBS comprised 137 NaCl, 2.7 KCl, 8.1 Na_2_HPO_4_, and 1.5 KH_2_PO_4_. The elastase solution was prepared by dissolving 0.1 % elastase in the control bathing solution. The Fura-2 AM solution comprised 12.5 μM Fura-2 AM (Molecular Probes, Eugene, OR) dissolved in Earle’s solution supplemented with 0.25 % Pluronic F-127 (Sigma). The paraformaldehyde solution was 4 % paraformaldehyde dissolved in PBS. The LY solution comprised 0.4 mg/ml LY (Sigma) dissolved in the 200 mM K^+^ bathing solution. The nominally Ca^2+^- and Mg^2+^-free solution comprised 150 NaCl, 5 KCl, 10 glucose, and 5 HEPES, which were buffered to pH 7.4 with NaOH.

## Results

### Cell type for the ACh-responding cells

**Fig. 1:**
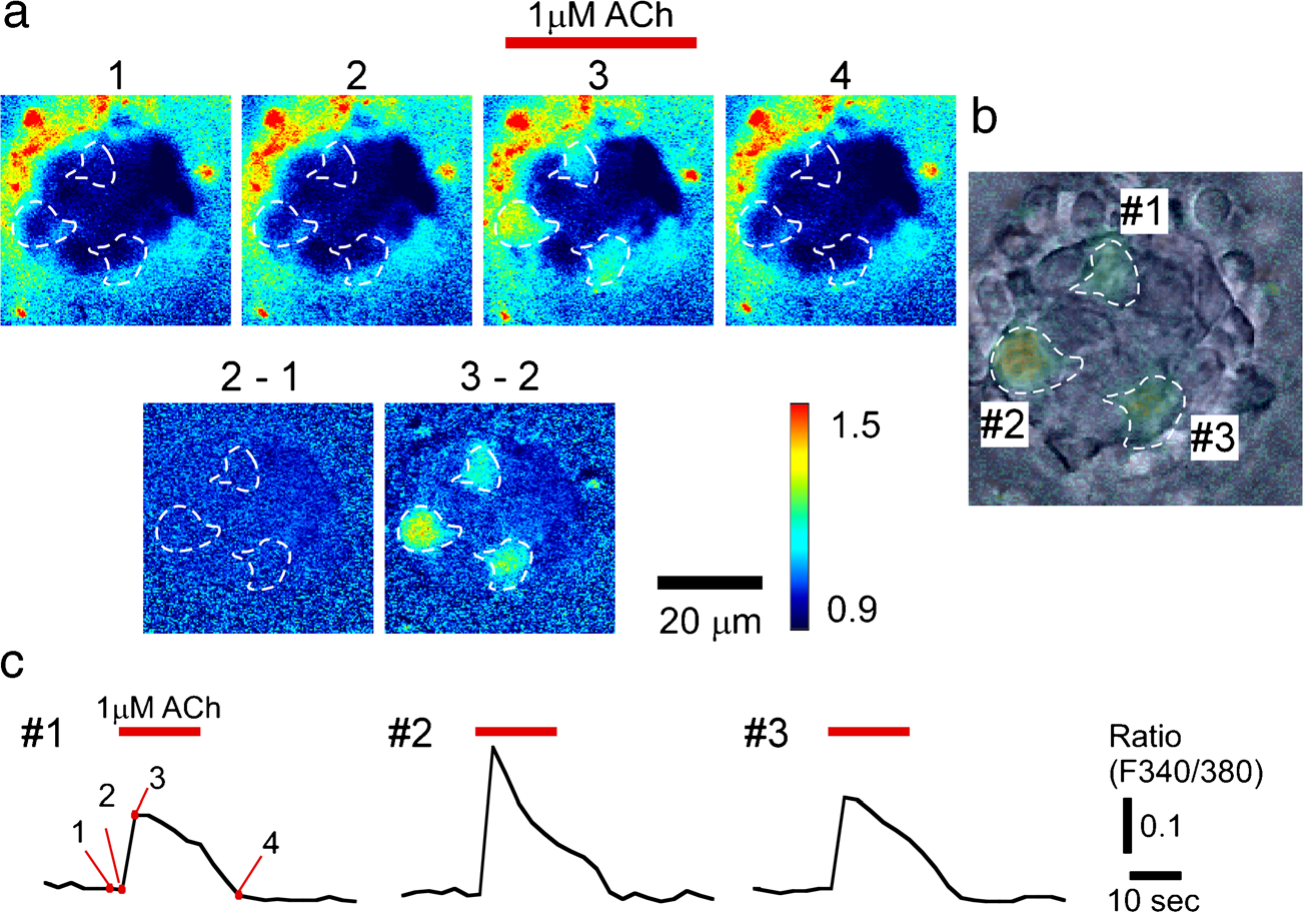
ACh-induced Ca^2+^ responses of TBCs. **a** Ca^2+^ imaging of a single taste bud (*upper row*) and their subtracted images (*lower row*, i.e., *3*–*2*: the subtraction of frame *2* from frame *3*). ACh (1 μM) was applied as shown in frame *3*. **b** Overlay of Ca^2+^ imaging of ACh-responding TBCs (*3*–*2*) in **a** and a differential interference contrast image of the taste bud. **c** Respective ACh-induced responses of the numbered TBCs in **b**. The *numerals* in trace no. 1 are identical to the frame numbers in **a**

**Fig. 2:**
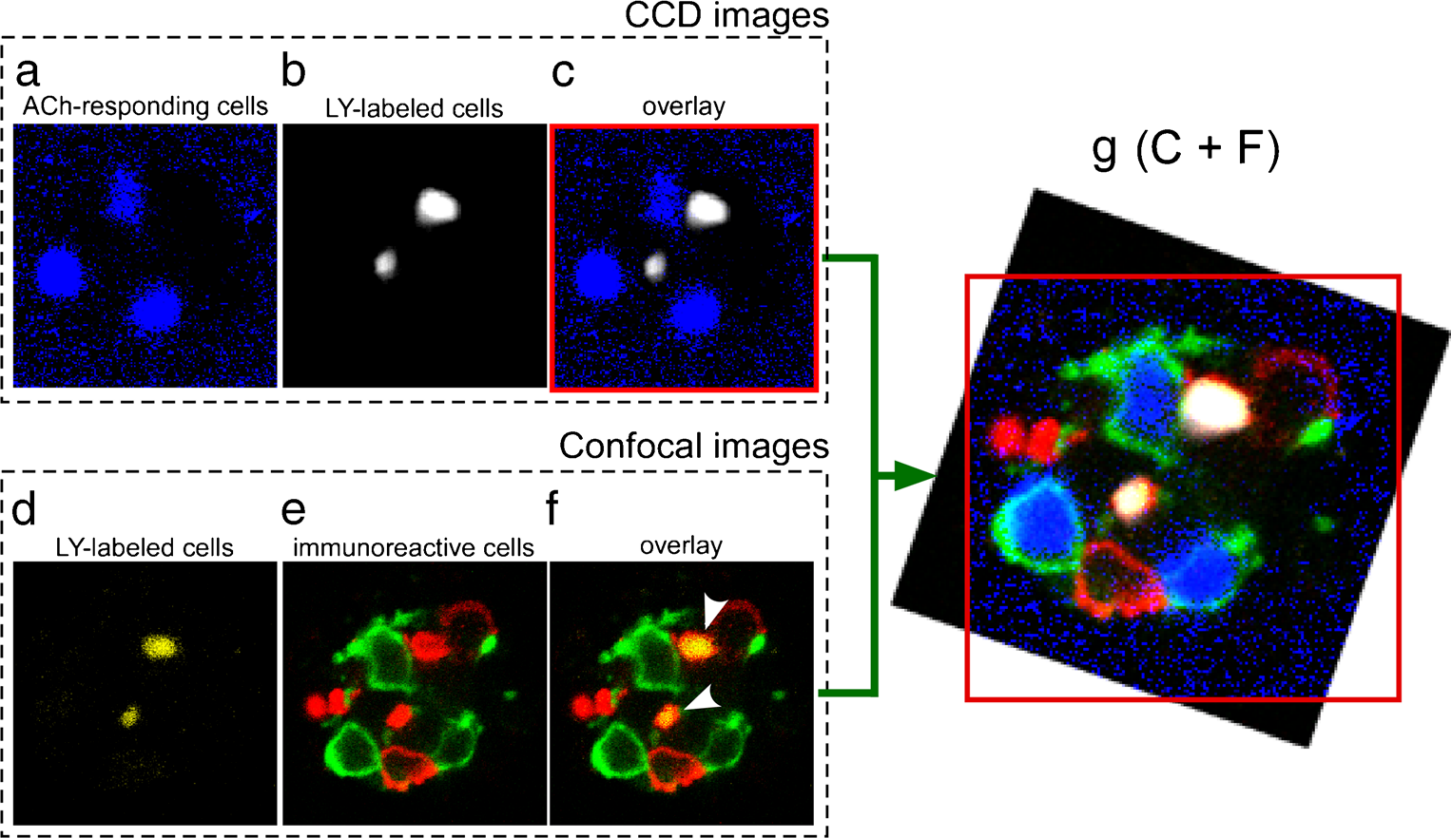
Positional relationships among ACh-responding cells, LY-labeled cells, and immunohistochemical cell types in the same taste bud. **a** Monochrome image of ACh-responding cells shown in Fig. 1a (*3*–*2*). **b** Image of LY-labeled cells. **c** Overlay with a *red outline* on the frame. **d** Confocal image of LY-labeled cells in the same taste bud shown in **a**–**c**. **e** Confocal image of immunoreactive cells. **f** Overlay. **g** Overlay of **c** and **f**, where **f** is rotated to superimpose LY-labeled cells. As shown by the *red outline*, the four corners of **c** have been eliminated. ACh-responding cells, *blue*; LY-labeled cells, *white* in images **b**, **c**, and **g**, and *yellow* in images **d**, **f**, and **g**; PLCβ2-immunoreactivity, *red*; SNAP-25-immunoreactivity, *green*. *Arrowheads* in **f** show the overlap of PLCβ2 and LY. It should be noted that images **d**–**f** were acquired after fixation for immunohistostaining (color figure online)

The application of 1 μM ACh dissolved in the control bathing solution to the basolateral membrane side transiently increased [Ca^2+^]_in_ in a few TBCs per taste bud, i.e., 2.4 ± 1.4 cells (mean ± SD, *n* = 14; Fig. 1a–c). Immediately after the Ca^2+^ imaging experiments were complete, we supravitally labeled type II cells in a taste bud with LY without moving the taste bud and investigated the positional relationships between the ACh-responding cells and the LY-labeled cells (Fig. 2a–c). We fixed the taste bud and immunohistochemically identified the cell type of the same TBC (Fig. 2d–f). The fluorescence of LY-labeled cells was still distinguishable after immunohistostaining. The overlay of the two positional relationships obtained by superimposing the LY-labeled cells in two positional relationships clearly showed that all of the ACh-responding cells were SNAP-25-immunoreactive (Fig. 2g).

Using this method, we investigated the cell types for 34 ACh-responding cells in 14 taste buds. All of the ACh-responding cells examined were immunoreactive to SNAP-25. PLCβ2-immunoreactive cells or non-immunoreactive cells did not respond to ACh. Not all of the SNAP-25-immunoreactive cells were ACh-responding cells, i.e., among 44 SNAP-25-immunoreactive cells found in these 14 taste buds, 10 (∼23 %) did not respond to ACh. These results showed that the ACh-responding cells comprised a subtype of the SNAP-25-immunoreactive cells.

### Ca^2+^ responses of ACh-responding cells to KCl-induced depolarization

Our results showed that the ACh-responding cells were immunoreactive to SNAP-25. SNAP-25-immunoreactive cells are identical to type III cells, and type III cells cause Ca^2+^ responses on depolarization because they express voltage-gated Ca^2+^ channels together with other voltage-gated channels [6, 24]. Therefore, we tested the depolarization-dependent Ca^2+^ responses of ACh-responding cells.

**Fig. 3:**
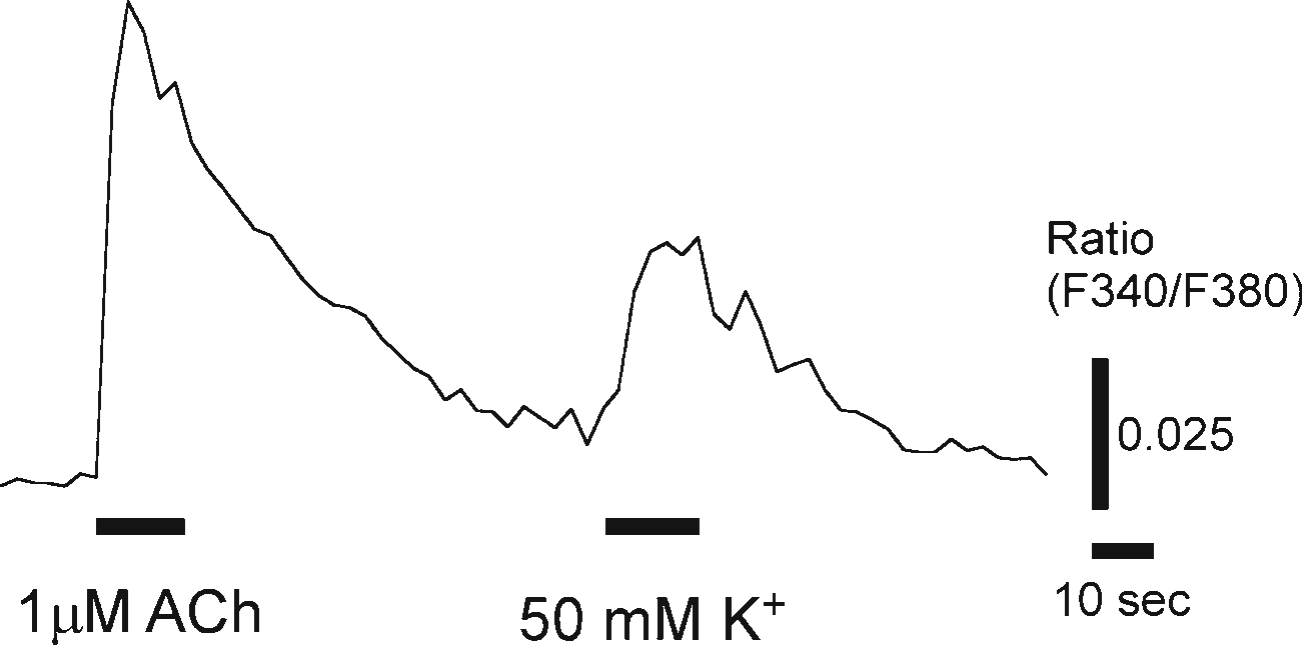
Ca^2+^ response of ACh-responding cells to 50-mM K^+^-induced depolarization. Representative trace of both ACh- and high K^+^-induced responses obtained from the same TBCs. *Bars* under the trace show the duration of ACh or KCl solution application as indicated.

Among 83 ACh-responding cells, 75 cells (90 %) responded with increased [Ca^2+^]_in_ due to the depolarization induced by the application of the 50 mM K^+^ bathing solution to their basolateral membranes (Fig. 3). Among 92 50 mM K^+^-responding cells, 75 cells (82 %) responded to ACh. These results suggest that most of the ACh-responding cells expressed voltage-gated Ca^2+^ channels.

### Transduction mechanism of the ACh response

**Fig. 4:**
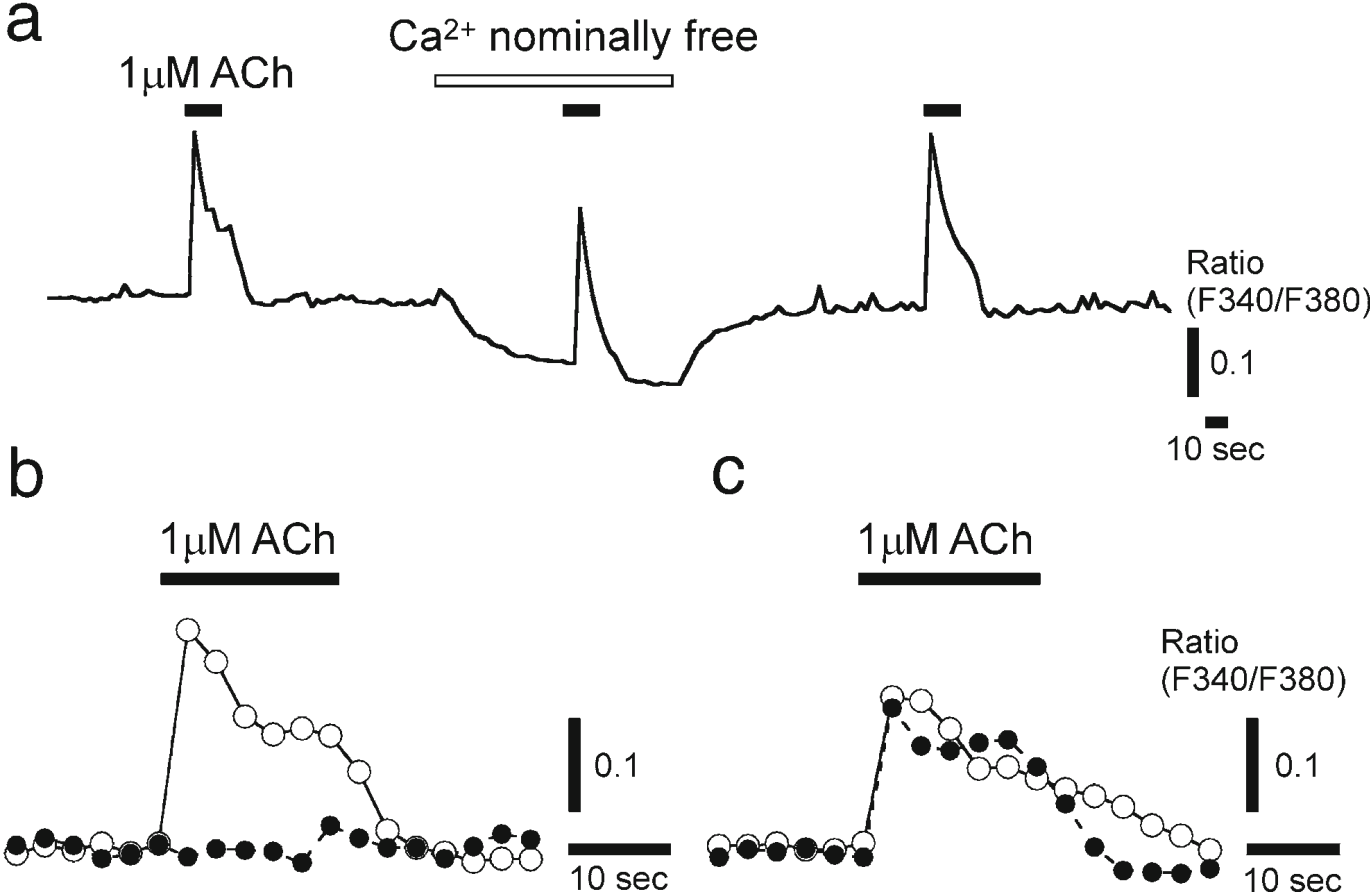
Effects of [Ca^2+^]_out_ and PLC inhibitors on the ACh-induced Ca^2+^ responses of single TBCs. **a** Representative Ca^2+^ responses in different [Ca^2+^]_out_. *Closed bars*, the duration of ACh application; *open bar*, duration of the application of nominally Ca^2+^-free solution. **b** Representative Ca^2+^ responses in the absence (*open circles*) and presence (*closed circles*) of 1 μM U73122. **c** Responses examined with 1 μM U73343, a negative control for U73122. Overlaid traces were obtained from the same single TBCs

The ACh-induced Ca^2+^ responses occurred in the nominally Ca^2+^-free bathing solution (Fig. 4a). The application of 1 μM U73122, a phospholipase C (PLC) inhibitor, to the basolateral membrane side significantly decreased the Ca^2+^ response to 0.31 ± 0.22 times the magnitude of the control response (*n* = 5, *p* < 0.008, one-tailed paired *t* test; Fig. 4b). Washing the taste bud with the control bathing solution failed to recover the magnitude of the response. The application of 1 μM U73343, a negative control, had no effects (0.90 ± 0.21, *n* = 5, *p* > 0.14 one-tailed paired *t* test; Fig. 4c).

These results show that in response to ACh, M3 caused the release of Ca^2+^ from intracellular stores via the activation of a PLC. Thus, we found that SNAP-25-immunoreactive cells expressed M3 and exhibited Ca^2+^ responses via a PLC cascade. This cascade never contains PLCβ2 and IP_3_R3, because type III cells are non-immunoreactive to them. We, therefore, searched for molecules involved in this cascade with RT-PCR by extracting mRNAs from whole taste buds, not single cells.

**Fig. 5:**
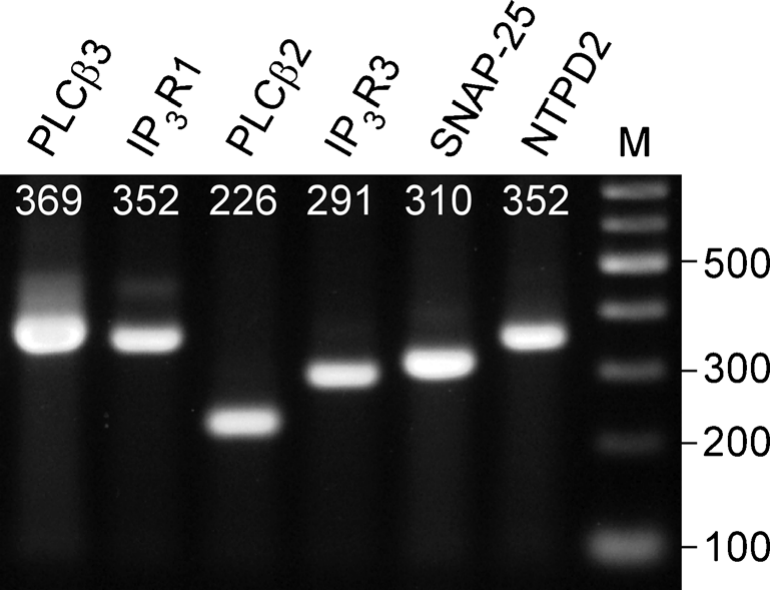
Expression of PLCβ3 and IP_3_R1 mRNAs in fungiform taste buds. Electrophoresis image of the PCR products. Isolated taste buds from mouse fungiform papilla expressed mRNAs for *PLCβ3* and *IP*_*3*_ *R1*, in addition to those for *PLCβ2*, *IP*_*3*_ *R3*, *SNAP-25*, and *NTPD2*. *Numerals* on each line indicate the expected product size. *M* molecular size marker

Our RT-PCR analyses detected mRNAs for PLCβ3 and IP_3_R1 in addition to PLCβ2 and IP_3_R3 in the fungiform taste buds (Fig. 5). The same results were obtained in duplicate experiments.

## Discussion

The results of the present study showed that all of the ACh-responding cells were immunoreactive to SNAP-25 and that 90 % of them increased [Ca^2+^]_in_ in response to 50-mM K^+^-induced depolarization. The type III cells were immunoreactive to SNAP-25 [35], and they expressed voltage-gated Ca^2+^ channels [6, 24]. Therefore, we conclude that all ACh-responding cells are type III cells.

Ten of 44 SNAP-25 immunoreactive cells (∼23 %, 0.7 ± 1.0 cells per taste bud) were insensitive to ACh. In addition, 8/85 ACh-responding cells (∼9 %) were insensitive to depolarization, and 17/95 50 mM K^+^-responding cells (∼18 %) were insensitive to ACh. These results show that the type III cells differed in terms of the expression of M3 and voltage-gated channels. Type III cells may comprise multiple subtypes.

Our results demonstrated that Ca^2+^ responses occurred in the nominally Ca^2+^-free bathing solution and that a PLC inhibitor, U73122, decreased the magnitude of the Ca^2+^ response. These results suggest that in response to ACh by type III cells, M3 activates an IP_3_ cascade that releases Ca^2+^ from intracellular stores. No type III cells were immunoreactive to Gα gustducin, PLCβ2, and IP_3_R3 [3, 6, 30]. However, our RT-PCR study detected mRNAs for PLCβ3 and IP_3_R1 in fungiform taste buds, thereby suggesting that type III cells functionally express these proteins. Their expression was also detected immunohistochemically in mouse IP_3_R3–GFP-negative cells [9]. Furthermore, members of the Gα q/11 family, such as Gα14 and Gα15, were detected in mRNA and protein forms [22, 31, 33]. Therefore, it is likely that type III cells or at least a subtype of them possess an IP_3_ cascade that differs from that of type II cells and that they release Ca^2+^ from intracellular Ca^2+^ stores in response to ACh.

The role of M3 in the taste response of type III cells remains unknown. Type III cells are known to sense acidic substances [14, 17, 36] and are considered to mediate the taste response of type II cells to taste nerve fibers [10, 13, 15]. It has been shown that M3-deficient mice are hypophagic and lean, which is probably due to the ingestion of lesser food than their wild-type littermates [34]. In addition, in islets, M3 has been shown to mediate the potentiation of glucose-induced insulin release via IP_3_ cascades [1, 7]. Therefore, it is possible that M3 contributes to the appropriate maintenance of the appetite by potentiating the release of neurotransmitters from type III cells.

The source of ACh release in taste buds is not fully understood, although ACh is a neurotransmitter released from the parasympathetic nervous system and is responsible for stimulating “rest-and-digest.” Immunohistochemical studies have shown that type II cells or trigeminal nerve fibers within the taste buds contain a vesicular ACh transporter [8, 28]. Also, the release of ACh from type II cells has been detected physiologically [5]. The mechanism of ACh release from type II cells remains to be investigated.

The results of the present study showed that M3 occurs only on type III cells but not on type II cells or non-immunoreactive cells. These results do not agree with those of a previous study, which demonstrated the presence of M3 on mouse type II cells [5]. This discrepancy may be due to differences in the materials and methods employed. In the present study, we used fungiform taste buds in situ and measured [Ca^2+^]_in_ with Fura-2 in the presence of 2 mM Ca^2+^. By contrast, the previous study used sliced circumvallate taste buds with calcium green dextran or calcium orange in the presence of 8 mM Ca^2+^. The Kd values of the Ca^2+^-sensing dyes employed were similar; hence, the discrepancy may be due to differences between the fungiform and the circumvallate taste buds. Furthermore, 8 mM Ca^2+^ was used, which is much higher than physiological Ca^2+^ concentrations, which may have resulted in the discrepancy.

In a previous paper [8], we discussed the possibility that M3 occurs on type II cells. However, the present results clearly show the selective expression of M3 on type III cells.
